# Household food insecurity and unimproved toilet facilities associate with child morbidity: evidence from a cross-sectional study in Bangladesh

**DOI:** 10.1186/s12889-022-13469-2

**Published:** 2022-05-31

**Authors:** Mohammad Ashraful Islam, Mahfuzur Rahman, Md. Fakhar Uddin, Md. Tariqujjaman, Gobinda Karmakar, Mohammad Ashikur Rahman, Matthew Kelly, Darren Gray, Tahmeed Ahmed, Haribondhu Sarma

**Affiliations:** 1 Nutrition and Clinical Services Division, icddr,b, Mohakhali Dhaka, 1212 Bangladesh; 2 Health, Nutrition and Population Programme, BRAC, Mohakhali Dhaka, 1212 Bangladesh; 3 Department of Global Health, National Centre for Epidemiology and Population Health, The Australian National University, Canberra, ACT 2021 Australia

**Keywords:** Household food insecurity, Sanitation, Under-five children; Bangladesh

## Abstract

**Background:**

Despite recent progress in rural economic development and food production, the prevalence of household food insecurity (FI) and use of unimproved toilet facilities are widespread in Bangladesh. Evidence regarding the consequencs of household FI and poor sanitation on child morbidity is scarce. This study aimed to understand the association of FI and unimproved toilet facility with morbidity status of under-5 children in Bangladesh.

**Methods:**

We used data from a cross-sectional survey that was conducted as part of an evaluation of the Maternal, Infant and Young Child Nutrition (MIYCN) Program in 9 districts of Bangladesh. The study population included children aged 6–59 months and their caregivers, identified using a two-stage cluster-sampling procedure. Child morbidity status was the outcome variable, and household FI status and type of toilet used were considered the main exposure variables in this study. We performed logistic regression, calculated adjusted odds ratios (AOR) to assess the association of child morbidity with household FI and unimproved toilet facility after adjusting for potential confounders.

**Results:**

A total of 1,728 households were eligible for this analysis. About 23% of the households were food-insecure, and a large number of households had improved toilet facilities (93.4%). In the multivariable logistic regression model, we found that children in food-insecure households with unimproved toilet facility had 5.88 (AOR: 5.88; 95% CI 2.52, 13.70) times more chance, of being morbid compared to the children of food-secure households with improved toilet facility. A similar association of FI and toilet facilities with each of the morbidity components was observed, including diarrhea (AOR:3.6; 95% CI 1.79, 7.89), fever (AOR:3.47; 95% CI 1.72, 6.99), difficult or fast breathing with cough (AOR:3.88; 95% CI 1.99, 7.59), and difficult or fast breathing with blocked or running nose (AOR:1.29; 95% CI 0.56, 2.95).

**Conclusions:**

Our study shows that household FI and unimproved toilet facility jointly have more deteriorative effects on child morbidity than either of these conditions alone. Therefore, it is recommended to consider these two critical factors while designing a public health intervention for reducing morbidity among under-five children.

**Supplementary Information:**

The online version contains supplementary material available at 10.1186/s12889-022-13469-2.

## Background

Household food insecurity (FI) is a measure of the availability of food in households and lack of access to an adequate amount of safe and nutritious food to fulfill dietary requirements for an active and healthy life [1]. FI is a multi-dimensional phenomenon that occurs in most countries at all income levels [2, 3], in 2020 globally 2.37 billion people remain food-insecure [3]. In 2020, the prevalence of experiencing severe FI was estimated at 60% in Africa, 26% in Asia, 50% in Latin America, and 9% in North America [3]. In Bangladesh, about 25% of the population remained FI in 2019 [4], despite the country making some progress in achieving food self-sufficiency through agricultural improvement, food production, as well as reducing under-five mortality [4]. According to the Global Food Security Index 2017, Bangladesh was ranked 89^th^ out of 113 countries for prevalence of food insecurity [5].

Previous studies have identified several adverse consequences of household FI among infants and young children [6], including long-term impacts on children’s health [7]. Studies conducted in low-income settings [6–10] observed significant associations between household FI and childhood illness. For example, a study in Ethiopia observed household FI as a significant risk factor for increasing morbidity. FI increased the risk of childhood diarrhea 1.44 times, cough 1.42 times and fever 1.53 times compared to children in food-secure households [8]. Children in FI households are also at risk of poor development, impaired performance in school and depression and poor health in adulthood, and are more likely to be stunted and suffer from undernutrition [11–13]. FI in children plays out through nutritional and non-nutritional pathways that lead to poor-quality diet (higher consumption of energy, fat, sugar, and fiber), less physical activity and their developmental consequences [14]. FI in children was significantly associated with iron deficiency anemia among infants and young children which indicate deleterious health, social, behavirorial and cognitive consequences for children [15].

As well, measuring FI alone may not be sufficient in assessing child morbidity-outcome risks if the other major driver of such morbidity, in terms of sanitation, is not considered [16]. Despite consistent improvement in rural economic development in Bangladesh, national survey data shows 57% of households were using unimproved toilet facilities in 2017 [17] and elsewhere a study showed not owning a toilet increased the likelihood of being a food insecure household [18]. The effect of unimproved toilet facilities on child undernutrition and childhood morbidity have been well established [19, 20]. A study conducted in rural northern Bangladesh observed that the use of unimproved toilet facilities increased the risk of childhood acute respiratory infection by 31% [21]. A study in India showed that access to an improved toilet can reduce childhood diarrhea by more than 2% [20]. Another recent study conducted in Myanmar observed children in the household with unimproved toilet facility were at significantly higher risk of suffering from cough and fever compared to households with improved toilets [22].

Although numerous studies have assessed the independent effect of household FI and unimproved toilet facilities on child morbidities [6–10], to our knowledge, no study has measured the combined effect of household FI and unimproved toilet facility on the morbidity status of children. This paper aimed to understand the association of FI and unimproved toilet with morbidities of under-5 children in Bangladesh. We expect that the findings of this study will inform the development of programs to improve child health outcomes through consideration of combinations of risk factors.

## Methods

### Data source

In this paper, we used the data from a cross-sectional survey that was conducted as part of an evaluation of the Maternal, Infant and Young Child Nutrition (MIYCN) Program of BRAC (formerly known as Bangladesh Rural Advancement Committee), an international non-governmental organization based in Bangladesh. Data was collected during April–May 2016.

### Study area

**Fig. 1:**
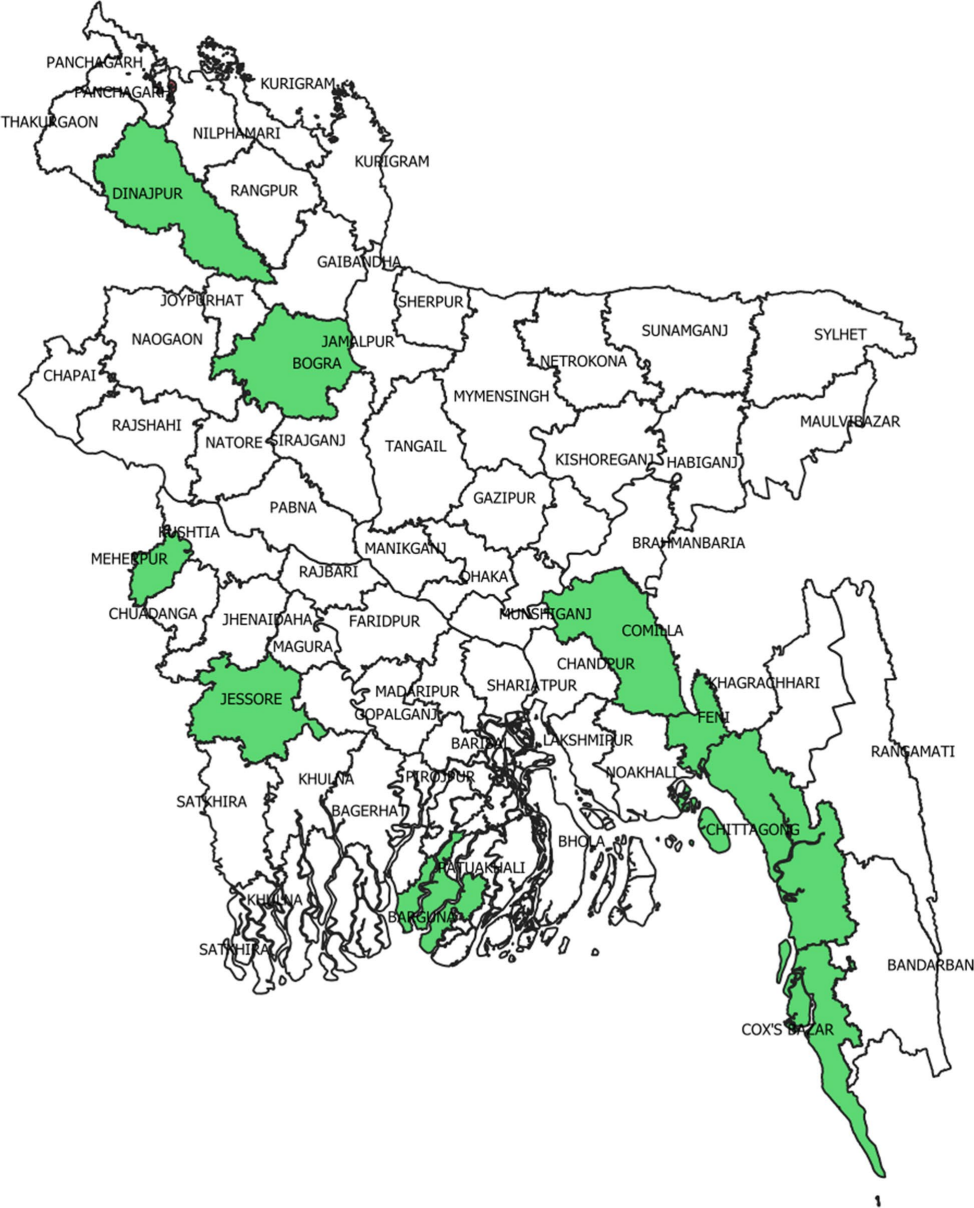
Map of Bangladesh–the highlighted districts indicate the study areas

The study area comprised nine districts including Barguna, Bogra, Chittagong, Comilla,Cox’s Bazar, Dinajpur, Feni, Jessore, Meherpur of Bangladesh where BRAC was implementing its MIYCN program (Fig. 1). BRAC has selected these districts considering the availability BRAC programme-delivery infrastructures including the availability of its community health workers who were trained to implement the MIYCN interventions at the community level.

### Study population

The study population included children aged 6–59 months and their caregivers. The caregiver was defined as the child’s biological mother or the person who takes care of or looks after and gives the child most meals on most days in the past seven days before the survey. Inclusion criteria allowed selection of the households of caregivers who had at least one child of 6–59 months and where the caregivers had resided in that household for at least one year. We excluded households if the caregiver was unable to attend an interview during the day of the survey due to illness or was unable to give consent to participate in the survey. If the household has more than one eligible child, we randomly selected one child for the survey.

### Sample-size and sampling

We calculated sample-size for a district-level estimation; we considered 50% prevalence, the precision of ± 10%, Zα value of 1.96, and a design effect of 2. The use of the standard sample-size calculation formula yielded the minimum sample-size of 192 households per district. Thus, the total sample-size was 1,728.

We followed a two-stage cluster-sampling procedure. In the first stage, systematic random- sampling was applied to select 16 Primary Sampling Units (PSUs) from the complete list of BRAC communities in a district. This procedure helped to ensure the equal chance of being included in the sample, and the resulting sample was close to an even spatial sample of BRAC’s target areas. In the second stage, the survey team ensured the population-size, total approximate households, and the boundaries of the PSU on arrival at the selected PSU and in consultation with and assistance of the local people (Union Parishad Chairman, Member, Counselor, school teacher, elderly person, and the relevant personnel of the locality). A physical map-segment sample approach was exercised to segment the selected community or PSU. The detailed sampling procedures have been reported elsewhere [23].

### Outcome variable

Child morbidity status was the outcome variable for this study. We considered children to be experiencing morbidity if their caregivers reported that their child had been sick either due to ailments, such as diarrhea (diarrhea with 3 or more loose or watery, bloody, pussy or mucous stools in a 24 h period), fever (illness with fever), illness with cough and had difficulty in breathing or fast breathing, difficult or fast breathing with blocked or runny nose in the last 14 days before the survey. In addition to the caregivers recall, we also collected child’s morbidity related information from the doctor’s prescriptions/medicines if the child received any treatment from a doctor during the last episode of illness. If caregivers reported any of the illnesses being present in their child, or if medical records indicated a sickness episode, then we considered them as experiencing morbidity.

### Exposure variable and covariates

Household FI status (categorized as food-insecure, food-secure) was considered the main exposure variable in this study. We assessed households FI status based on 9 questions (Additional file 1) from the Household Food Insecurity Access Scale (HFIAS) developed by the Food and Nutrition Technical Assistance (FANTA) group in collaboration with Tufts University and Cornell University [24]. The response to each question ranges from 0 to 30. We made scoring of these responses as 0 = 0, 1–2 = 1, 3–10 = 2, and 11–30 = 3. The total score ranged from 0 to 27 for 9 questions. We then categorized as score 0–1 = food-secure household and 2–27 = food-insecure household. Other exposure variables included toilet availability, categorized as: Improved toilet (flush or pour flush to a piped sewer system, septic tank, pit latrine, Kumasi Ventilated Improved Pit latrine, pit latrine with slab), unimproved toilet (pit latrine without a slab, hanging latrine or defecate in bush or field). We also combined food security and toilet facilities of households to see the combined effects in regression analysis and we categorized them as: Food-secure and improved toilet, Food-secure and unimproved toilet, Food-insecure and improved toilet, Food-insecure and unimproved toilet.

Other covariates included household-size (categorized as: < 5, ≥ 5), number of 6–59 months old children in the household (categorized as: one, two, or more), child’s age (6–23 months, 24–59 months), any children in the household aged 5–14 year who were attending school, caregiver’s age (< 25 years, ≥ 25 years), caregiver’s education (< 5 years, ≥ 5 years), father’s age (categorized as: < 30 years, ≥ 30 years), caregiver’s religion (categorized as: Muslim, Hindu/Other religion), caregiver’s occupation (categorized as: other, housewife), wealth index (categorized as: poor, middle, rich), and monthly household income [categorized as: < 11,000 BDT (Bangladeshi taka), ≥ 11,000 BDT (83 BDT = 1 USD)]. A supplementary file on response categories and variable description has been described about the variables used in this study (Supplementary table 1).

### Data collection

The study was conducted in accordance with the National/International human ethics guidelines and regulations. Before data collection all participants provided written informed consent. To collect data, we formed a survey team of four members, including two interviewers, a medical technologist, and a supervisor. At the time of recruitment of team members, priority was given to the members who were experienced and/or involved in the previous surveys. The supervisor was mainly responsible for selection of the study participants, using the mentioned sampling methods, monitoring the data-collection activities, and ensuring the quality of the data by spot-checking and re-interviewing.

We measured the level of anxiety and uncertainty of the participants about household food supply, insufficient quality of food, and insufficient food intake by following the HFIAS that comprises a brief survey instrument to assess whether households have experienced problems with accessing food during the last 30 days of survey. The questionnaire used a nine-item household hunger scale questionnaire [(i) worry about food, (ii) unable to eat preferred foods, (iii) eat just a few kinds of foods, (iv) eat foods they really do not want to eat, (v) eat a smaller meal, (vi) eat fewer meals in a day, (vii) no food of any kind in the household, (viii) go to sleep hungry, and (ix) go a whole day and night without eating)].

Before finalizing the questionnaire, a field test was conducted in a real-field setting in the non-survey areas, and the feedback from the field test was incorporated into the final version of the questionnaire. It was then submitted to the Institutional Review Board (IRB) of icddr,b for review and approval. A Standard Operating Procedure (SOP) was developed for the interviewers. This SOP was a guide for the interviewers on how to ask each of the questions to the participants. The electronic data-collection procedures used an Android-based Smartphone program of survey questionnaire. To support the Android operating system, Open Data Kit (ODK) software was used for developing the program. TABs/Smartphones were used and both Bangla and English versions questionnaire were used in the ODK software.

### Data analysis

Weighted and cluster (PSU)-adjusted descriptive statistics were estimated and presented in percentages with respective 95% confidence intervals. Bivariate analysis using a chi-square test was performed to measure the association between the outcome variable (morbidity status of the children) and main exposure variables (household FI and toilet facilities). We performed multivariable logistic regression analysis to measure the association between outcome variables and other independent variables. At first, we performed unadjusted logistic regression to find the significant variables for the final multivariable regression model; *p*-value of < 0.05 was considered for the significance level. Finally, multivariable logistic regression was performed to assess the association of child morbidity with household FI and unimproved toilet facility after adjusting for potential confounders and presented in adjusted odds ratios with a 95% confidence interval. All analyses were performed using statistical software STATA (Version 13).

## Results

Socio-demographic and background characteristics of the study participants have been presented in Table 1. A total of 1,728 households were eligible for this analysis. Around 61% of households had ≥ 5 members, and about 85% had one child aged 6–59 months of age. Most of the selected respondents were biological mothers (97%) of the eligible children. Caregivers aged > 25 years were 55 and 76.2% of the caregivers had completed ≥ 5 years of schooling. About 68% of fathers of the eligible children completed five or more years of schooling.

**Table 1:** Socio-demographic and background characteristics by morbidity status of the study participants

Indicator	*N* = 1,728% (n)	Morbidity status		*p*-value
		**Yes, % (n)**	**No, % (n)**	
Household members				
< 5	39.4 (680)	56.8 (368)	43.2 (312)	0.616
≥ 5	60.6 (1048)	55.4 (578)	44.6 (470)	
Age (years) of mother/caregiver				
≤ 25 years	45.1 (805)	53.8 (424)	46.2 (381)	0.136
> 25 years	54.9 (923)	57.7 (522)	42.3 (401)	
Mother’s/Caregiver’s with ≥ 5 years of education				
No	23.9 (399)	60.3 (236)	39.7 (163)	0.097
Yes	76.2 (1329)	54.6 (710)	45.4 (619)	
Religion of respondents				
Hindu and other	8.3 (174)	47.8 (84)	52.2 (90)	0.092
Muslim	91.7 (1554)	56.7 (862)	43.3 (692)	
Most recent birth of caregiver				
> 12 months	83.4 (1438)	55.0 (767)	45.0 (671)	0.141
≤ 12 months	16.6 (290)	60.5 (179)	39.5 (111)	
Working status of caregiver				
Other	6.3 (106)	58.0 (58)	42.0 (48)	0.714
Housewife	93.7 (1622)	55.8 (888)	44.2 (734)	
Age (years) of fathers				
≤ 30 years	41.1 (714)	54.2 (379)	45.8 (335)	0.231
> 30 years	58.9 (999)	57.3 (559)	42.7 (440)	
Fathers with ≥ 5 years of education				
No	32.3 (543)	58.1 (308)	41.9 (235)	0.281
Yes	67.8 (1185)	54.9 (638)	547 (45.1)	
Age categories of child (in months)				
6–23 months	39.7 (685)	60.6 (408)	39.4 (277)	0.011
24–59 months	60.3 (1043)	52.9 (538)	47.1 (505)	
Sex of children				
Female	47.0 (822)	55.0 (436)	45.1 (386)	0.533
Male	53.0 (906)	56.8 (510)	43.2 (396)	
Children aged 5–14 years attending school				
No	2.9 (28)	62.4 (19)	37.6 (9)	0.599
Yes	97.1 (1097)	57.7 (620)	42.3 (477)	
Number of children 6–59 months				
One	85.3 (1477)	54.9 (783)	45.1 (694)	0.105
Two and above	14.7 (251)	62.0 (163)	38.0 (88)	
Food security status				
Food-insecure household	23.1 (387)	66.5 (253)	33.5 (134)	0.001
Food-secure household	76.9 (1341)	52.8 (693)	47.2 (648)	
Toilet facilities of household				
Improved toilet	93.4 (1602)	74.6 (88)	25.4 (38)	0.001
Unimproved toilet	6.6 (126)	54.6 (858)	45.4 (744)	
Combined food security and toilet facilities of households				
Food-secure and improved toilet	73.7 (1272)	52.4 (654)	47.6 (618)	< .001
Food-secure and unimproved toilet	3.3 (69)	62.0 (39)	38.0 (30)	
Food-insecure and improved toilet	19.7 (330)	63.0 (204)	37.0 (126)	
Food-insecure and unimproved toilet	3.4 (57)	86.8 (49)	13.2 (8)	
Wealth Index				
Poor	21.2 (345)	52.2 (183)	47.8 (162)	0.338
Middle	40.4 (705)	57.2 (401)	42.8 (304)	
Rich	38.5 (678)	56.7 (362)	43.3 (316)	
Income of household				
< BDT 11,000	53.2 (926)	58.9 (525)	41.1 (401)	0.021
≥ BDT 11,000	46.8 (802)	52.6 (421)	47.5 (381)	

*p*-values were generated from Chi-square test

Sixty percent of the children belonged to the age-group of 24–59 months. Around 97% of the children aged 5 to 14 years were attending school. The ratio of boys and girls was 53:47. Fifty-six percent of caregivers of under-5 children reported that their children had been sick in the last two weeks prior to the day of the interview. Results showed that about 23% of the households were food-insecure, and a large number of households had improved toilet facilities (93.4%).

Table 2 presents the association of morbidity status with food security and toilet facility status of the households. There was a significant (*p* < 0.001) association between morbidity status and either FI or unimproved toilet facility in the household. In food-secure households, 52.8% of children were found with morbidity but, in food-insecure households, child morbidity prevailed among 66.5% of households (*p* < 0.001). About 55% of children from households that had improved toilet facility had morbidity whereas this figure was about 75% in the households that had unimproved toilet facilities (*p* < 0.001). In the unadjusted logistic regression model, we found that the children of food-insecure households had 77% (OR: 1.77; 95% CI 1.27–2.47) more likelihood of being morbid compared to children of food-secure households. Children of households with unimproved toilet facilities had 2.44 (OR: 2.44; 95% CI 1.46–4.06) times higher chances to be morbid compared to children of households with improved toilet facilities **(**Table 2).

**Table 2:** Association between household food security and availability of toilet facilities and morbidity status of children who participated in the study

**Indicator**	**Any morbidity**		***p*-value**	**OR**	**95% CI**	**AOR**^a^	**95% CI**
	**Yes, n (%)**	**No, n (%)**					
Food security status							
Secure	52.8 (693)	47.2 (648)	< 0.001	1		1	
Insecure	66.5 (253)	33.5 (134)		1.77***	1.27–2.47	1.60***	1.15–2.22
Toilets facilities							
Improved toilet facilities	54.6 (858)	45.4 (744)	< 0.001	1		1	
Unimproved toilet facilities	74.6 (88)	25.4 (38)		2.44***	1.46–4.06	2.08***	1.24–3.48

^a^The model adjusted by caregiver’s education, caregiver’s religion, number of children aged 6–59 months, and household income

^*^p < 0.05, **p < 0.01, ***p < *0.001* (using Z- test)

Chi-square test reported row percentage (*p*-values of less than 0.05 were regarded as statistically significant)

In multivariable logistic regression, we adjusted the model with caregiver’s education, caregiver’s religion, number of children aged 6–59 months in the household, household income, household food security status, and toilet facility of the household; it was found that children of food-insecure households were 60% (AOR: 1.60; 95% CI 1.15, 2.22) more likely to be morbid compared to children of food-secure households (Table 2). Furthermore, children of households with unimproved toilet facilities had 2.08 (AOR: 2.08; 95% CI: 1.24, 3.48) times higher likelihood of being morbid compared to children of households with improved toilet facilities (Table 2).

**Fig. 2:**
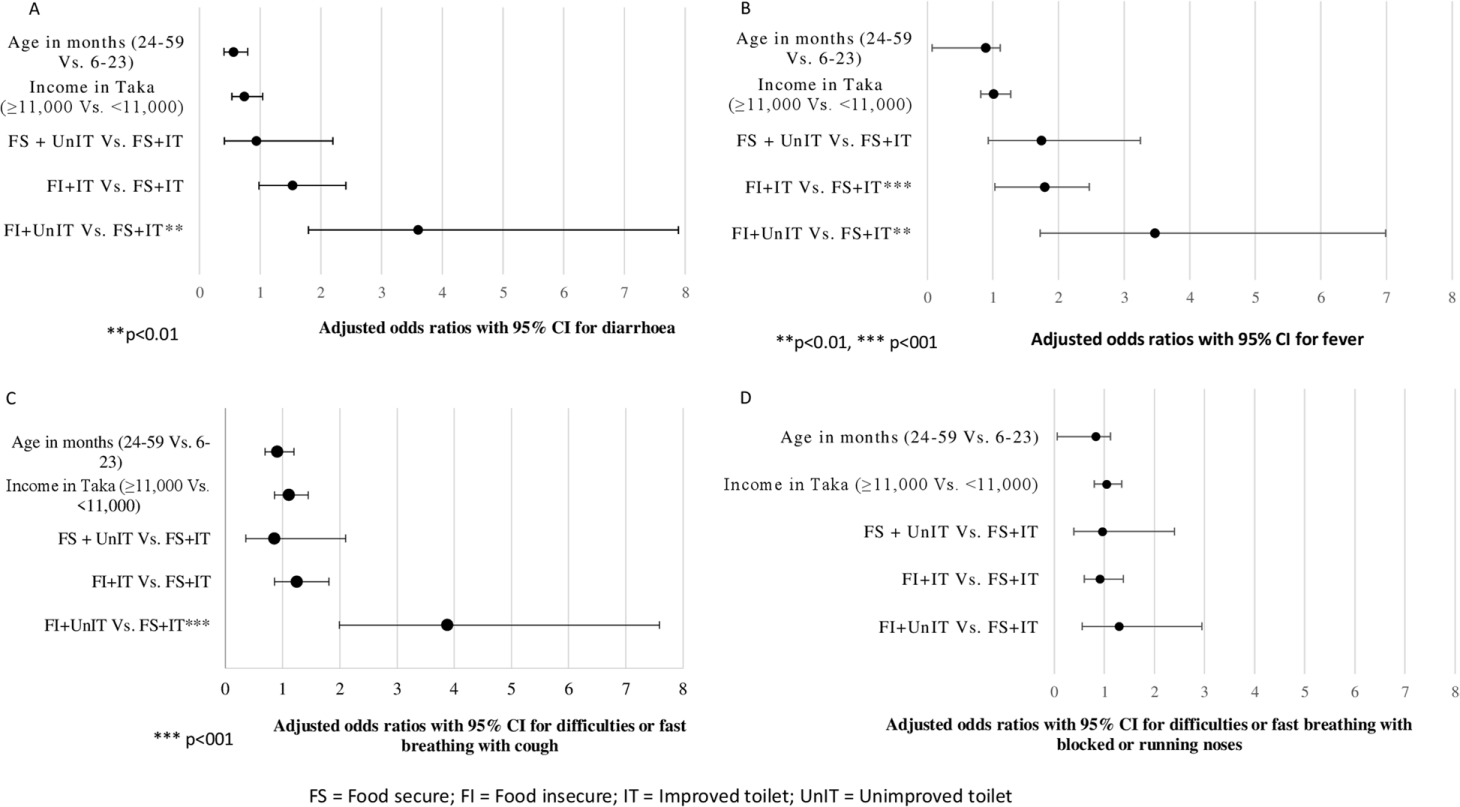
Household FI and unimproved toilet facilities associated with each of the four morbidity statuses of children

The simple and multivariable logistic regression model to explore the associated factors of morbidity status is presented in Table 3. In the unadjusted logistic regression model, we found age of children, household income, and combination of food security and toilet facility to be significantly associated with morbidity status of under-five children. In the multivariable logistic regression model, we considered all significant variables of the simple logistic regression model and found that children of food-insecure households with improved toilet facility had 53% (AOR: 1.53; 95% CI 1.09, 2.15) more chance, and food-insecure children with unimproved toilet facility had 5.88 (AOR: 5.88; 95% CI 2.52, 13.70) times more chance, of being morbid compared to the children of food-secure households with improved toilet facility (Table 3). A similar association of food security and toilet facilities with each of the morbidity components was observed, including diarrhea (AOR:3.6; 95% CI 1.79, 7.89), fever (AOR:3.47; 95% CI 1.72, 6.99), difficult or fast breathing with cough (AOR:3.88; 95% CI 1.99, 7.59), and difficult or fast breathing with blocked or running nose (AOR:1.29; 95% CI 0.56, 2.95) (Fig. 2).

**Table 3:** Factors associated with morbidity status of children who participated in the study

Independent variable	OR	95% CI	AOR^¶^	95% CI
Household members				
< 5	1			
≥ 5	0.94	0.74–1.19		
Age (years) of mothers/caregiver				
≤ 25 years	1			
> 25 years	1.17	0.95–1.44		
Mothers/Caregivers with ≥ 5 years of education				
No	1			
Yes	0.79	0.60–1.04		
Religion of respondents				
Hindu and other	1			
Muslim	1.43	0.94–2.17		
Most recent birth of caregiver				
> 12 months	1			
≤ 12 months	1.25	0.93–1.69		
Working status of caregiver				
Other	1			
Housewife	0.92	0.57–1.47		
Age (years) of father				
≤ 30 years	1			
> 30 years	1.13	0.92–1.40		
Father with ≥ 5 years of education				
No	1			
Yes	0.88	0.70–1.11		
Category of child’s age (in months)				
6–23 months	1		1	
24–59 months	0.73*	0.57–0.93	0.70**	0.55–0.90
Sex of child				
Female	1			
Male	1.08	0.85–1.37		
Children aged 5–14 years attending school				
No	1			
Yes	0.82	0.39–1.72		
Number of children aged 6–59 months				
One	1			
Two and above	1.34	0.94–1.92		
Wealth Index				
Poor	1			
Middle	1.22	0.96–1.56		
Rich	1.20	0.89–1.61		
Income of household				
< BDT11000	1		1	
≥ BDT 11,000	0.77*	0.62–0.96	0.89	0.72–1.10
Combined food security and toilet facilities of households				
Food-secure and improved toilet	1		1	
Food-secure and unimproved toilet	1.48	0.81–2.73	1.49	0.79–2.83
Food-insecure and improved toilet	1.55*	1.11–2.16	1.53*	1.09–2.15
Food-insecure and unimproved toilet	5.97***	2.66–13.37	5.88***	2.52–13.70

^*^p < 0.05, **p < 0.01, ***p < 0.001

## Discussion

The findings of this study revealed that household FI and unimproved toilet facility have independent effects on child morbidity. However, FI and unimproved toilet facility combined can have a more severe effect than either alone. These findings have important implications for the development of programmes to alleviate common childhood diseases. Some other studies have reported the association between FI and general health consequences [25–27]; studies also have shown that poor general health, mental and physical health have a relationship with FI [28, 29]. Studies conducted in Brazil and Colombia found that household FI and child morbidity, such as diarrheal disease among the children, were significantly associated [3, 9] corresponding to the findings of our study.

The interventions or programs taken so far in low- and middle-income countries are vertical and specific to address one particular problem: either FI or child morbidity. Their relationship is often overlooked while designing an intervention. Nevertheless, an integrated program could be cost-effective targeting both improving food security and sanitation status of the households, and, thus, to reduce child morbidity. A prevous study considered both household FI and adequate access to water and sanitation behaviour suggesting the consideration of integrated interventions to reduce acute malnutrition and childhood morbidity among children [30]. A study in Ethiopia showed that Integration of WASH and nutrition was associated with less stunting and disease in children 0–59 months in a setting with poor WASH conditions [31]. That was also supported by the recent analysis showing integrating antihelminth treatement with the home fortification with micornutrients powders reduced anaemia among children in Bangladesh [32].

It is very likely that the food-insecure households have poor living conditions. The unimproved toilet is one of the dimensions of poor living conditions. Studies indicate that the use of unimproved toilets increases the burden of diseases, like diarrhea among poor children [10, 33]. A recent study conducted in Cameroon suggested that children from households practicing open defecation and/or having poor hygiene practices had higher chance of developing diarrhea [30].Our study also found that the children belonging to food-insecure households having unimproved toilet facilities are more likely to suffer from diarrhoea, fever, and fast breathing with cough. Therefore, the study corroborates with the suggested potential nutrition-sensitive interventions such as social protection and safety net program to improve food security and living conditions of the households leading to improvement of child health [34].

Other studies also show that younger children are more susceptible to morbidity when compared to their older counterparts [35]. Apart from household FI and unimproved toilet facility, child’s age is also found to be associated with morbidity in our study. Childhood morbidity, particularly diarrheal diseases, are more prevalent among the younger children compared to the older ones [36]. Since child’s age is a non-modifiable factor, the study suggests undertaking of interventions targeting the younger children for reducing morbidity, irrespective of household food security and living conditions.

### Limitations and strengths

Being a community-based study; the participation rate was considerably high as the respondents were at the households during the day of the interview. The survey was cross-sectional and, so, it was difficult to assess the causality between the outcome and exposure variables. Morbidity assessments were self-reported by children caregivers through recall of the preceding two weeks from the date of the interview; so, there was a possibility of recall bias. FI data were collected through recall, and the situation prevailed in the past 30 days. The accuracy of data depended on the respondent’s memory and honesty. To overcome this, the interviewers were given special training to increase the ability to understand the questions to be asked to the respondents. Self-reported family income and expenditure could also be subject to bias. In the multiple regression model, we didn't include the wealth index variable. We included only three variables, child's age, household income and combined food security and toilet facilities of households, which were found significant in the simple regression model. However, we also checked the multicollinearity among these three variables and found the mean VIF (variance inflation factor) is 1.07 and the individual VIF ranges from 1.00 to 1.10, which indicating the negligible collinearity among the independent variables.

## Conclusions

Our study shows that household FI and unimproved toilet facility jointly have more deteriorative effects on child morbidity than either of these conditions alone. Unlike other studies, this illustrates the independent and combined effects of household FI and unimproved toilet facility on child morbidity. Taking measures on food security at the household level may not be sufficient to develop the nutritional status of children, rather an integrated programme on food security and sanitation is recommended to improve the overall health outcomes of the children. This evidence could show the pathway for the implementers and policy-makers that intervention targeting the improvement of the health status of children should not only consider the food security status of the households but also the sanitation condition of the households.

## Supplementary Information

**Additional file 1.** Household Hunger Scale questionnaire.

**Additional file 2:** **Supplementary Table 1.** Questions, responses and variables description used in this paper.

## Data Availability

The data that support the findings of this study are available from corresponding author (HS) upon reasonable request and with permission of icddr,b IRB.

## Abbreviations

FI: Food insecurity
MIYCN: Maternal, Infant and Young Child Nutrition
AOR: Adjusted odds ratios
BRAC: Bangladesh Rural Advancement Committee
PSU: Primary Sampling Units
HFIAS: Household Food Insecurity Access Scale
FANTA: Food and Nutrition Technical Assistance
BDT: Bangladeshi taka
