# Supplementary material for: Household food insecurity and unimproved toilet facilities associate with child morbidity: evidence from a cross-sectional study in Bangladesh
Source: BMC Public Health. 2022 May 31;22:1075. doi: 10.1186/s12889-022-13469-2 (PMC9158165; doi:10.1186/s12889-022-13469-2)
Supplement: Supplementary file 1 — Additional file 1. Household Hunger Scale questionnaire. [file 12889_2022_13469_MOESM1_ESM.docx]

**Additional file 1.** Household Hunger Scale questionnaire

| **Household Hunger Scale (Food Security)** | |
| --- | --- |
| Ques. No. | **Questions** |
| Q.1 | In the past 4 weeks (30 days), how often did you worry that your household would not have enough food days? |
| Q.2 | In the past 4 weeks (30 days), how often were you or any household member not able to eat the kinds of foods that you or s/he preferred because of a lack of resources? |
| Q.3 | In the last 4 weeks (30 days), how often did you or any household member have to eat a limited variety of foods due to a lack of resources? |
| Q.4 | In the last 4 weeks (30 days), how often did you or any household member have to eat some foods that you or s/he really did not want to eat because of a lack of resources to obtain other types of food? |
| Q.5 | In the last 4 weeks (30 days), how often did you or any household member have to eat a smaller meal than you or s/he felt was needed because there was not enough food? |
| Q.6 | In the last 4 weeks (30 days), how often did you or any other household member have to eat fewer meals in a day because there was not enough food? |
| Q.7 | In the last 4 weeks (30 days), how often was there ever no food to eat of any kind in your household because of lack of resources to get food? |
| Q.8 | In the last 4 weeks (30 days), how often did you or any household member go to sleep at night hungry because there was not enough food? |
| Q.9 | In the last 4 weeks, how often did you or any household member go a whole day and night without eating anything because there was not enough food? |
