# Supplementary material for: Household food insecurity and unimproved toilet facilities associate with child morbidity: evidence from a cross-sectional study in Bangladesh
Source: BMC Public Health. 2022 May 31;22:1075. doi: 10.1186/s12889-022-13469-2 (PMC9158165; doi:10.1186/s12889-022-13469-2)
Supplement: Supplementary file 2 — Additional file 2: Supplementary Table 1. Questions, responses and variables description used in this paper. [file 12889_2022_13469_MOESM2_ESM.docx]

**Supplementary Table 1:** Questions, responses and variables description used in this paper

| **Variable details in the questionnaire** | **Value lebel** | **The variable name used in this study** | **Description/categorization** |
| --- | --- | --- | --- |
| How many people live in your household? |  | Household members | We categorized as <5 members and ≥5 based on the national cut-off. |
| Mother/ caregiver Age (years) | in years | Age (years) of mother/caregiver | We categorized as <25 years and ≥25 years based on the median value. |
| Mother/ caregiver have 5 or more years of education? | Yes=1 No=2 | Mother’s/Caregiver’s with ≥5 years of education | We categorized mothers/caregiver’s educational status as she had ≥5 years of education (yes) or less than five years of education (no). |
| What is your religion? | 1.Hindu 2. Islam  3.Christian 4. Buddhist  5.None, 6. Prefer not to say 7. Other | Religion of respondents | Respondents religious status were categorized as “Muslim” and “Hindu & others” based on the percentage distribution of different categories. |
| When was your most recent birth? | in months | Most recent birth of caregiver | We categorized most recent birth (in months) as >12 months and ≤12 months based on literature review. |
| Mother/ caregiver Occupation | 1.Housewife 2.Worker 3.Unemployed 4. Service  5. Business 6.Farming 7.Tailor 8. Others specify | Working status of caregiver | We categorized mothers/caregivers working status as “Other” and “Housewife” based on the percentage distribution of different categories. |
| Father Age (years) | in years | Age (years) of fathers | We categorized fathers age as <30 years and ≥30 years based on the median value. |
| Fathers have 5 or more years of education? | Yes=1 No=2 | Fathers with ≥5 years of education | We categorized father’s educational status as he had at least five years of education or less than five years of education. |
| child Age (months) | in months | Age categories of child (in months) | We categorized the child age (in month) as 6-23 months and 24-59 months based on the literature review. |
| child Sex | (M=1 / F=2) | Sex of children | Child sex were categorized as Male and female. |
| Any children of aged 5–14 years are not currently attending school? | Yes=1 No=2 Not- Applicable=777 | Children aged 5-14 years attending school | We categorized 5 to 14 years children who currently attending school as “yes” and “no”. |
| How many children with 6-59 months age in your household? | (If there are more than 1 eligible children then randomly select one from them and go on) | Number of children 6-59 months | Number of children 6-59 months were categorized as “one and two” & “above” based on the literature review. |
| What is the total monthly income of your household? (last one month) | in taka | Income of household | We categorized households income as <BDT 11000 and ≥BDT 11000 based on the median value. |
| **Food security status** |  |  |  |
| In the past 4 weeks (30 days), how often did you worry that your household would not have enough food? days? | times/days | Food security status | We categorized as “food secure” and “insecure” based on the HFIAS sacle. |
| In the past 4 weeks (30 days), how often were you or any household member not able to eat the kinds of foods you preferred because of a lack of resources? | times/days |  |  |
| In the last 4 weeks (30 days), how often did you or any household member have to eat a limited variety of foods due to a lack of resources? | times/days |  |  |
| In the last 4 weeks (30days), how often did you or any household member have to eat some foods that you really did not want to eat because of a lack of resources to obtain other types of food? | times/days |  |  |
| In the last 4 weeks (30 days), how often did you or any household member have to eat a smaller meal than you felt you needed because there was not enough food? | times/days |  |  |
| In the last 4 weeks (30 days), how often did you or any other household member have to eat fewer meals in a day because there was not enough food? | times/days |  |  |
| In the last 4 weeks (30 days), how often was there ever no food to eat of any kind in your household because of lack of resources to get food? | times/days |  |  |
| In the last 4 weeks (30 days), how often did you or any household member go to sleep at night hungry because there was not enough food? | times/days |  |  |
| In the last 4 weeks, how often did you or any household member go a whole day and night without eating anything because there was not enough food? | times/days |  |  |
| **Morbidity status** |  |  |  |
| Diarrhea with 3 or more loose or watery, bloody, pussy or mucous stools in a 24-hour period? | Yes=1 No=2 | Morbidity status | We categorized as “yes” if a child presents any symptoms of diseases mentioned in the questionnaire, otherwise “no” . |
| illness with fever? | Yes=1 No=2 |  |  |
| illness with cough and had difficulty in breathing or fast breathing | Yes=1 No=2 |  |  |
| was the fast or difficult breathing due to a problem in the blocked or running nose? | Yes=1 No=2 |  |  |
| No illness happened | Yes=1 No=2 |  |  |
| **Toilet facilities of household** | |  |  |
| What kind of toilet facility do members of your household usually use? | Piped sewer system | Toilet facilities of household (improved) | We categorized toilet facilities of the households as improved (if household have Piped sewer system, Septic tank, Pit latrine, KVIP latrine, Pit latrine with slab) and otherwise defined as unimproved. |
|  | Septic tank | Toilet facilities of household (improved) |  |
|  | Pit latrine | Toilet facilities of household (improved) |  |
|  | KVIP latrine | Toilet facilities of household (improved) |  |
|  | Pit latrine with slab | Toilet facilities of household (improved) |  |
|  | Composting toilet | Toilet facilities of household (unimproved) |  |
|  | Elsewhere (not specified above) | Toilet facilities of household (unimproved) |  |
|  | Pit latrine without slab | Toilet facilities of household (unimproved) |  |
|  | Bucket | Toilet facilities of household (unimproved) |  |
|  | Hanging latrine | Toilet facilities of household (unimproved) |  |
|  | Bush or field No facilities | Toilet facilities of household (unimproved) |  |
|  | Other | Toilet facilities of household (unimproved) |  |
|  |  |  |  |
| **Wealth index** |  | Wealth index | We constructed the wealth index using principal component analysis and categorized as poor, middle and rich. |
| Does your household have electricity? | Electricity |  |  |
| What fuels does your household use for cooking? | Gas |  |  |
|  | Kerosene |  |  |
|  | Biogas (Gobar gas) |  |  |
|  | Solar |  |  |
|  | Coal lignite |  |  |
|  | Charcoal |  |  |
|  | Wood/Bamboo |  |  |
|  | Straw/shrubs/grass/crop residue |  |  |
|  | Animal dung |  |  |
|  | Other |  |  |
|  | Dirt/earth/sand/dung / mixed = 1 |  |  |
| Main material of the floor of the dwelling? | Other (tiles, concrete, wood) =2 |  |  |
|  | At least 15 decimal cultivable Land? |  |  |
| Does your household have...? | A television? |  |  |
|  | A mobile or non-mobile telephone? |  |  |
|  | Cattle/poultry |  |  |
|  | A motorcycle, bike, scooter, rickshaw, rickshaw-van, boat with engine? |  |  |
|  | A car, truck, jeep, or tractor? |  |  |
|  | A refrigerator? |  |  |
|  | A computer /laptop /tablet |  |  |
|  | A horse /cow /donkey /buffalo cart? |  |  |
|  | Furniture and related items |  |  |
|  | Agro-machineries |  |  |
|  | Other |  |  |
